# Serological profile of foot-and-mouth disease in wildlife populations of West and Central Africa with special reference to *Syncerus caffer* subspecies

**DOI:** 10.1186/s13567-015-0213-0

**Published:** 2015-07-08

**Authors:** Antonello Di Nardo, Geneviève Libeau, Bertrand Chardonnet, Philippe Chardonnet, Richard A Kock, Krupali Parekh, Pip Hamblin, Yanmin Li, Satya Parida, Keith J Sumption

**Affiliations:** Institute of Biodiversity, Animal Health and Comparative Medicine, College of Medical, Veterinary and Life Sciences, University of Glasgow, Glasgow, United Kingdom; The Pirbright Institute, Pirbright, Surrey, Woking United Kingdom; Centre de Coopération Internationale en Recherche Agronomique pour le Développement (CIRAD), UMR Contrôle des Maladies, Campus International de Baillarguet, Montpellier, France; African Protected Areas & Wildlife Expert, Saint Cloud, France; International Foundation for the Conservation of Wildlife (IGF), Paris, France; Royal Veterinary College (RVC), Hatfield, United Kingdom; European Commission for the Control of Foot-and-Mouth Disease (EuFMD), Food and Agriculture Organisation of the United Nations (FAO), Rome, Italy; Present address: Boehringer Ingelheim GmbH, Shanghai, China

## Abstract

The role which West and Central African wildlife populations might play in the transmission dynamics of FMD is not known nor have studies been performed in order to assess the distribution and prevalence of FMD in wild animal species inhabiting those specific regions of Africa. This study reports the FMD serological profile extracted from samples (*n* = 696) collected from wildlife of West and Central Africa between 1999 and 2003. An overall prevalence of FMDV NSP reactive sera of 31.0% (216/696) was estimated, where a significant difference in seropositivity (*p* = 0.000) was reported for buffalo (64.8%) as opposed to other wild animal species tested (17.8%). Different levels of exposure to the FMDV resulted for each of the buffalo subspecies sampled (*p* = 0.031): 68.4%, 50.0% and 0% for Nile Buffalo, West African Buffalo and African Forest Buffalo, respectively. The characterisation of the FMDV serotypes tested for buffalo found presence of antibodies against all the six FMDV serotypes tested, although high estimates for type O and SAT 3 were reported for Central Africa. Different patterns of reaction to the six FMDV serotypes tested were recorded, from sera only positive for a single serotype to multiple reactivities. The results confirmed that FMDV circulates in wild ruminants populating both West and Central Africa rangelands and in particular in buffalo, also suggesting that multiple FMDV serotypes might be involved with type O, SAT 2 and SAT 1 being dominant. Differences in serotype and spill-over risk between wildlife and livestock likely reflect regional geography, historical circulation and differing trade and livestock systems.

## Introduction

Foot-and-mouth disease (FMD) is an economically devastating disease of intensive livestock farming and high production animals, caused by a virus member of the *Apthovirus* genus within the *Picornaviridae* family, and characterised by an acute and highly contagious vesicular disease which can develop into persistent infection. Vesicular lesions resulting from FMD infection are mainly found in tongue, lips and feet but in some cases lesions also can occur in snout, muzzle, teats, skin and rumen. The disease is characterised by a very short incubation period and high level of virus excretion, particularly in pigs [1]. In wildlife, the FMD pathogenesis varies from a completely inapparent to a rare acutely lethal infection, making the diagnosis difficult either because the variability in the severity of presenting clinical signs is greater than in domestic livestock or because it tends to be subclinical for the particular species/virus combination [2,3]. The transmission dynamic of FMD in sub-Saharan Africa is mainly driven by two epidemiological cycles: one in which wildlife plays a significant role in maintaining and spreading the disease to other susceptible wild and/or domestic ruminants [4-6], whilst with the second the virus is solely transmitted within domestic populations and hence is independent of wildlife. More than 70 wild animal species have been demonstrated to be susceptible to the FMD virus (FMDV) either by natural infection or by experimental challenge, and on several occasions the virus has been isolated from naturally infected animals [7]. Among these, Cape buffalo (*Syncerus caffer caffer*) has been clearly shown to serve as long-term maintenance host (i.e. carrier) for the Southern African Territories (SAT) FMDV serotypes [8-10], and in populations of Cape buffalo the virus has been estimated to persist for 24 years or longer [11]. Infection in buffalo is subclinical and normally occurs in calves as soon as maternal antibodies wane at 2–6 months of age. Acutely infected buffalo provide sources of infection for other ruminants, both domestic and wild, directly or through other species which have contracted the infection from buffalo [5,6]. Although the implication of the buffalo carriers in the epidemiology of FMD has not been fully clarified, they have so far been shown to transmit the disease while in that state [4,8,12]. Phylogenetic relationships between SAT types FMDV strains isolated from cattle and those carried by buffalo have been reconstructed from different area of southern Africa, proving that contacts between livestock and buffalo regularly result in FMD outbreaks among cattle [13,14]. Furthermore, available evidence based on FMDV genome sequencing indicates that impala (*Aepyceros melampus*) populations of the Kruger National Park – South Africa, usually become infected with SAT viruses derived from buffalo [5]. On occasions SAT lineages were demonstrated to have been transmitted first from buffalo to impala and then from impala to cattle [6,15]. Conceived as such, impala can provide a conduit of infection between buffalo and livestock, acting as an important intermediate between domestic and wild ruminants and as an amplifying host in the context of FMD transmission dynamics [16]. Presence of antibodies against the FMDV in several wildlife species has been documented in studies conducted in different countries of the African continent, but mainly within its eastern and southern regions [16-18]. In addition, serological screenings implemented in East African countries have indicated potential infections of Cape buffalo with A and O FMDV serotypes [19-23], although current data do not support the primary role of buffalo and other wild animal species in the transmission of those FMDV serotypes generally occurring in domestic ruminants. This represents an important pattern of the FMD transmission dynamics in large parts of sub-Saharan Africa that still remains to be explained. Much is already known about the role that Cape buffalo plays in the FMD epidemiology, largely from studies conducted in South and East Africa; conversely, knowledge on the relationship between FMDV and wildlife and/or other buffalo subspecies that populate the rangelands of western and central African regions has been less thorough. In order to progress in the knowledge gap of the FMD epidemiology in sub-Saharan Africa and to further investigate the role of wildlife in the transmission of FMD, in this study the prevalence of antibodies against the FMDV nonstructural protein (NSP) and serological profiles of six out of the seven FMDV serotypes have been reconstructed from wildlife samples collected from national parks and faunal reserves of West and Central Africa. The aims of this study were: firstly, to produce an overall picture of the FMD prevalence in wildlife and mainly in buffalo subspecies of West and Central Africa, also characterising risk factors likely associated with the observed prevalence; secondly, to identify the FMDV serotypes potentially circulating in resident buffalo populations within the study area. In addition, potential limitations of diagnostic testing procedures used have been evaluated.

## Materials and methods

### Study population

**Figure 1:**
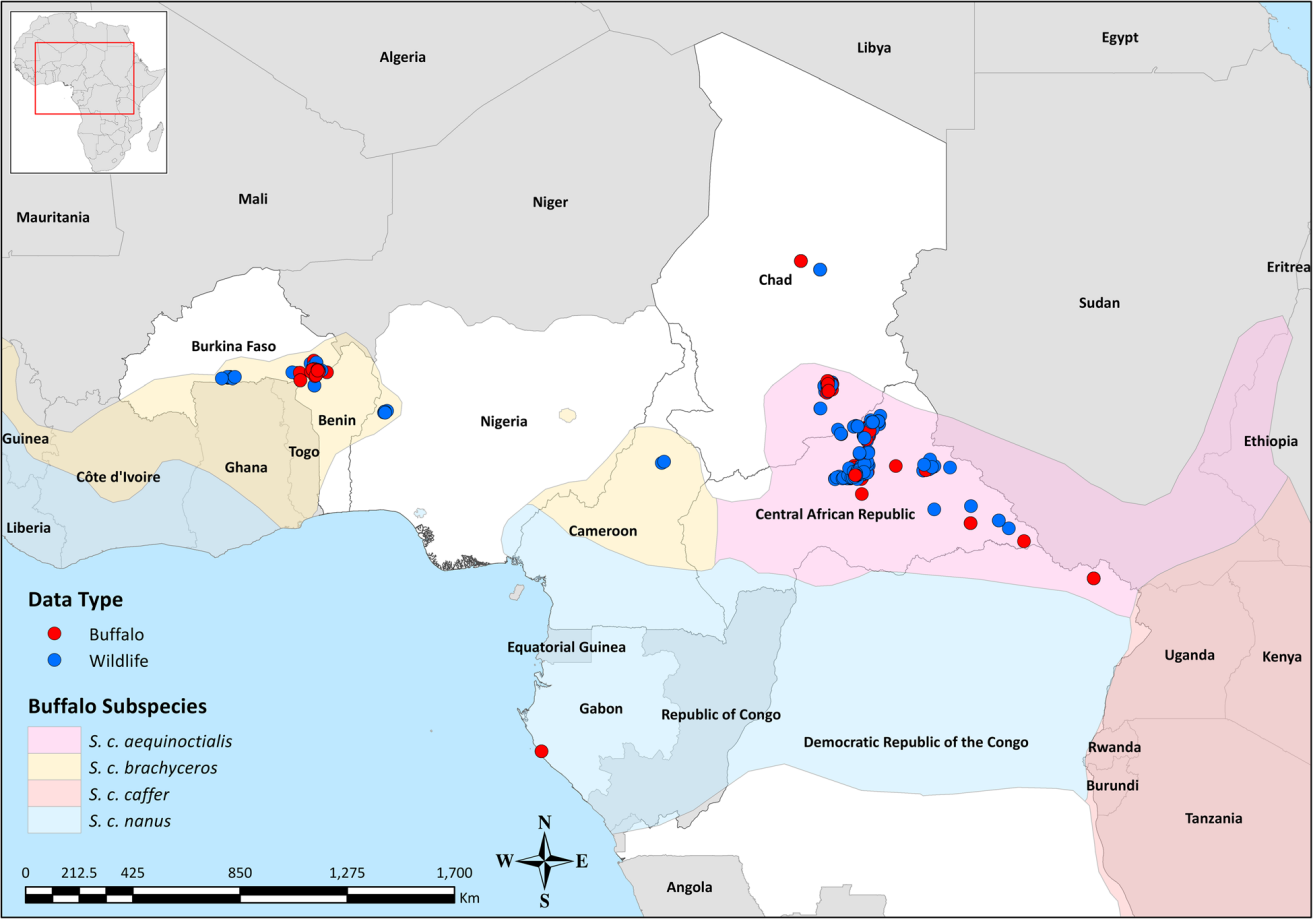
**Geographical locations of the wildlife samples selected by species.** Distributional extents of buffalo subspecies of the *Syncerus* genus sourced and adapted from [68].

The study was undertaken on serum samples collected from wild ruminants and pigs species during the African Wildlife Veterinary Project [24], as part of the Pan-African Rinderpest Campaign (PARC) and the subsequent programme for the Pan-African Control of Epizootics (PACE) implemented in 34 countries across the African continent between 1986 and 2007. Wildlife species and sampling sites were selected at the time according to susceptibility to the Rinderpest (RP) virus, population biology (i.e. richness, gregarious behaviour, and seasonal movements), interface between livestock and wildlife, and their availability for veterinary interventions. Sampling was performed using purposive sampling by immobilisation, opportunistic sampling by cropping and/or hunting and during field investigations of reported episodes of disease and mortality. From the whole collection stored at the Centre de Coopération Internationale en Recherche Agronomique pour le Développement (CIRAD), Montpellier – France, 696 sera collected between 1999 and 2003 were selected as representative of wildlife populations present in West and Central Africa (Table 1; Figure 1). In addition, further 19 samples collected from cattle within the transfrontier area of the Central African Republic and Chad were included for comparative purpose. Extracted aliquots were sent to The Pirbright Institute, Pirbright – United Kingdom (UK), for diagnostic testing.

**Table 1:** Wildlife samples tested allocated by region, country and species of collection.

**Region**	**Country**	**No. Buffalo Samples**	**No. Other Wildlife**	**TOT**
**West Africa**	Benin	18	11	29
	Burkina Faso	5	30	35
	Nigeria	1	7	8
	**TOT**	24	48	72
**Central Africa**	Cameroun	-	2	2
	Central African Republic	81	247	328
	Chad	53	203	256
	Democratic Republic of Congo	34	-	34
	Gabon	4	-	4
	**TOT**	172	452	624
**TOT**		196	500	696^†^

^†^Not including the 19 samples collected from cattle.

### Testing methodology

The sample collection was initially screened for antibodies against the highly conserved NSP of the FMDV using the PrioCHECK® FMDV NS Enzyme-Linked Immunosorbent Assay (ELISA) test kit (Prionics AG, Switzerland), according to the manufacturer protocol [25]. Specifically, a positive result was considered with a Percentage of Inhibition (PI) value of ≥50, whereas a strong positive result was set at a PI value of ≥70. Subsequently, the NS ELISA positive reactive sera were further assessed using the Solid Phase Competition ELISA (SPCE) *in-house* test developed at The Pirbright Institute – UK [26,27], thus enabling the qualitative and quantitative characterisation of the specific antibody responses to 6 of the 7 FMDV serotypes (A, O, C, SAT 1, SAT 2 and SAT 3) and, therefore, the FMDV serotyping profile of each of the serotypes present in Africa at the time of the sampling. The cut-off for the SPCE was set at a PI value of ≥50 for serotypes A, O and C, whilst a value of ≥40 PI was set for SATs serotypes [26]. Since the SPCE has not been validated for testing wildlife sera, the data were further reassessed increasing the cut-off to a value of ≥60 PI for serotypes A, O and C, and of ≥50 PI for SATs serotypes to account for an unpredicted high false positive response. These additional cut-offs were set as the mean of the single negative response of each serotype +5SD as estimated from the original validation data [26]. According to the above defined thresholds, a strong positive response was thus considered at either a PI value of ≥70 or ≥80. Results from the SPCE were confirmed selecting a random sub-sample of the resulting SPCE positive reactive sera by Virus Neutralization Test (VNT), as prescribed by the World Organisation for Animal Health [28], and using the O_1_ Manisa, A_22_ Iraq 24/64, C Phi 7/84, SAT 1 105, SAT 2 Eritrea, SAT 3 309 FMDV strains. Cut-off for positivity with the VNT was set at a titre of ≥1:45, whereas a titre of ≤1:11 and >1:11 but ≤1:32 were considered as negative and inconclusive, respectively.

### Data analysis

The original database stored at the CIRAD and consisting of information collected through paper forms during the field campaigns was manipulated and inspected for missing and/or illogical data entries and completed and/or corrected whenever possible. The ELISA results were stored in an Access 2010 (Microsoft Corporation) database along with associated metadata, such as geographical location and GPS coordinates, national park and date of collection, species and age. Statistical analyses were performed in R 3.1.2 [29], where confidence intervals were calculated using the Agresti-Coull estimation of binomial proportions [30]. Univariate analysis was carried out by the Adjusted-Wald test, considering the effect of species, age, year of sampling, location and park area on FMD seroprevalence [31]. All statistically significant variables (*p* < 0.05 two-sided) in the univariate analysis were further assessed by a generalised linear model (GLM) with a *logit* function using a stepwise selection approach, in order to characterise potential risk factors associated with the observed FMD seroprevalence. The probability of FMD seroprevalence μ was then calculated by back-transforming the estimated *logit* values *g*(*x*) as $\mu =\frac{e^{g(x)}}{1+{e}^{g(x)}}$ [32] and then introduced in a geospatial analysis environment using ArcGIS 10.2.2 (Environmental System Research Institute, Inc.) to produce a kernel smoothed intensity map of the predicted FMD prevalence [33]. Pairwise correlation analysis based on the Pearson’s product–moment coefficient (ρ) was undertaken on all possible combinations of PI estimates resulted from the SPCE testing [34], where missing data were treated as pairwise deletions.

## Results

**Figure 2:**
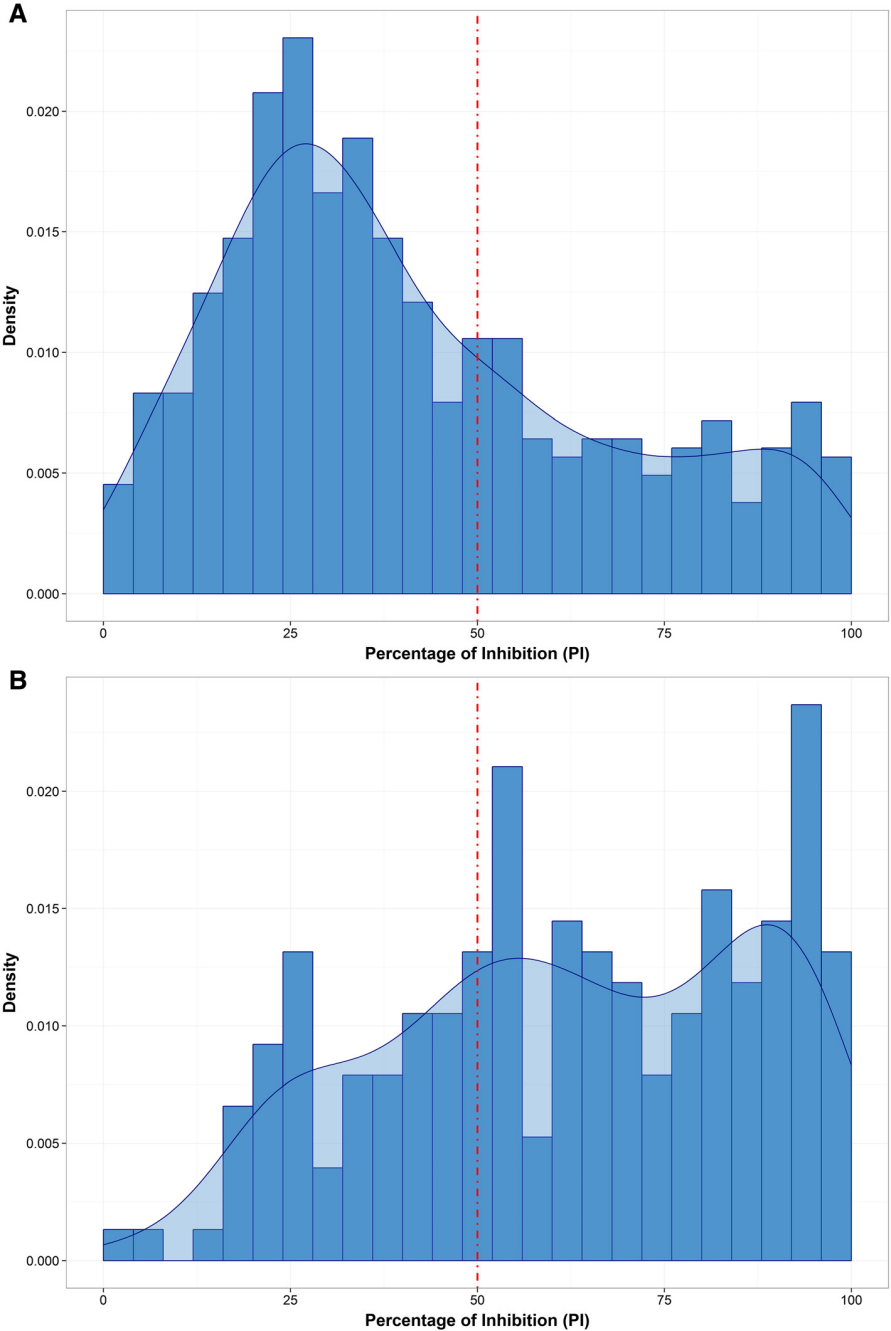
**Histogram and kernel density plots of the NS ELISA percentage of inhibition values estimated for the complete dataset (A) and for buffalo only (B).** Red dash-dot line sets the cut-off point (PI = 50).

The distributions of PI values resulted from the NS ELISA test for all the wildlife species (A) and for the buffalo samples only (B) are plotted in Figure 2. The 50th percentile for all species was reported as 34.2 PI (95%CI 32.0 – 36.3), different from the buffalo distribution that returned a value of 62.0 PI (95%CI 55.1 – 66.8). This figure would reflect a defined distinction between the seronegative (mainly non-buffalo species) and the seropositive (mainly buffalo) populations, confirmed by the bimodal distribution found for all species and the left-skewed distribution for the buffalo only. In addition, a total of 39 out of 89 (43.8%) positive samples for the non-buffalo species were found having a PI value of ≥70 in contrast with the 61.4% (78/127) estimated for the buffalo population. Thirteen out of 19 samples (68.4%) tested positive for cattle, confirming potential previous exposure of domestic livestock to the FMD; 84.6% (11/13) of those were returning PI values of ≥70.

### FMD seroprevalence in wildlife species: descriptive and univariate analyses

The NSP testing of the non-buffalo wildlife reported FMD positivity in 89 out of 500 samples (17.8%, 95%CI 14.7% – 21.4%), extracted from 16/27 species (59.3%). Among all the wildlife species assessed, presence of antibodies were found in individuals belonging to the *Alcelaphinae* (8.0%), *Antilopinae* (2.1%), *Bovinae* (14.3%), *Cephalophinae* (23.5%), *Hippotraginae* (7.1%), and *Reduncinae* (27.2%) sub-families (*Bovidae* family, 18.4%), and for species belonging to the *Suidae* family (11.6%). Bohor Reedbuck (*Redunca redunca*) (66.8%), Defassa Waterbuck (*Kobus ellipsiprymnus unctuosus*) (63.2%), Red-Flanked Duiker (*Cephalophus rufilatus*) (60.0%), Giant Eland (*Taurotragus derbianus*) (21.4%) and Topi (*Damaliscus korrigum jimela*) (21.4%) were the wild ruminants reporting high levels of FMD seropositivity (Table 2). The only sample collected from hippopotami and tested positive to the NS ELISA (PI = 53) should be regarded as a false positive response to the NS ELISA test, recalling that previous studies conducted in the Kruger National Park failed to detect antibodies against the FMDV in this species [2].

**Table 2:** Observed prevalence of FMDV NSP antibodies reported for all the wildlife species tested.

**Specie**	**No Positive/TOT**	**Observed seroprevalence**	**95% CI**
African Bush Elephant (*Loxodonta Africana*)	0/1	0%	-
African Forest Buffalo (*Syncerus caffer nanus*)	0/4	0%	-
Blue Duiker (*Philantomba monticola*)	1/5	20.0%	2.0% - 64.0%
Bohor Reedbuck (*Redunca redunca*)	4/6	66.7%	29.6% - 90.7%
Bongo (*Tragelaphus eurycerus*)	0/2	0%	-
Buffon’s Kob (*Kobus kob*)	24/172	13.9%	9.5% - 20.0%
Bush Duiker (Sylvicapra grimmia)	0/5	0%	-
Bushbuck (*Tragelaphus scriptus*)	2/15	13.3%	2.5% - 39.1%
Common Warthog (*Phacochoerus africanus*)	4/29	13.8%	4.9% - 31.2%
Defassa Waterbuck (*Kobus ellipsiprymnus unctuosus*)	36/57	63.2%	50.1% - 74.5%
Dorcas Gazelle (*Gazella dorcas*)	0/40	0%	-
Giant Eland (*Taurotragus derbianus*)	3/14	21.4%	6.8% - 48.3%
Giant Forest Hog (*Hylochoeurs meinertzhageni*)	0/1	0%	-
Greater Kudu (*Tragelaphus strepsiceros*)	0/4	0%	-
Hartebeest (*Alcelaphus buselaphus*)	1/20	5.0%	0% - 25.4%
Hippopotamus (*Hippopotamus amphibious*)	1/1	100%	-
Kordofan Giraffe (*Giraffa camelopardis antiquorum*)	0/5	0%	-
Lelwel Hartebeest (*Alcelaphus buselaphus lelwel*)	2/40	5.0%	0.5% - 17.4%
Nile Buffalo (*Syncerus caffer aequinoctialis*)	115/168	68.4%	61.1% - 75.0%
Nolan Warthog (*Phacochoerus africanus africanus*)	0/7	0%	-
Oribi (*Ourebia ourebi*)	1/7	14.3%	0.5% - 53.3%
Red River Hog (*Potamochoerus porcus*)	1/6	16.7%	1.1% - 58.2%
Red-Flanked Duiker (*Cephalophus rufilatus*)	3/5	60.0%	22.9% - 88.4%
Red-Fronted Gazelle (*Eudorcas rufifrons*)	0/1	0%	-
Roan Antelope (*Hippotragus equinus*)	2/28	7.1%	0.9% - 23.7%
Tiang (*Damaliscus korrigum korrigum*)	1/5	20.0%	2.0% - 64.0%
Topi (*Damaliscus korrigum jimela*)	3/14	21.4%	6.8% - 48.3%
West African Buffalo (*Syncerus caffer brachyceros*)	12/24	50.0%	31.4% - 68.6%
Western Hartebeest (*Alcelaphus buselaphus major*)	0/8	0%	-
Yellow-Backed Duiker (*Cephalophus silvicultur*)	0/2	0%	-
**TOT Buffalo**	127/196	64.8%	57.9% - 71.1%
**TOT Other Wildlife**	89/500	17.8%	14.7% - 21.4%
**TOT Cattle**	13/19	68.4%	45.8% - 84.8%

Adjusted-Wald test F_(29, 667)_ = 10.06 (*p* = 0.000).

Effect of the region of collection was found to be of statistical significance on seroprevalence estimates (*p* = 0.003), where 19% of samples tested positive for Central Africa whilst presence of antibodies were detected in only few samples collected in West Africa (3/48). Filtering the results by country of origin, a high FMD prevalence was estimated in samples obtained from Chad (23.1%), although these data were obtained from mostly a single national park (Zakouma National Park), thus likely reflecting local conditions.

### FMD seroprevalence in buffalo subspecies: descriptive and univariate analyses

In total, 127 out of 196 tested positive for FMD (64.8%, 95%CI 57.9% – 71.1%). According to the subspecies of the *Syncerus* genus, a high level of NSP antibodies was reported in both Nile Buffalo (*Syncerus caffer aequinoctalis*) (68.4%) and West African Buffalo (*Syncerus caffer brachyceros*) (50.0%) (Table 3). Although only few samples were tested (0/4), no FMDV reactive sera were found for African Forest Buffalo (*Syncerus caffer nanus*). The difference in seroprevalence observed between each of the buffalo sub-species was reported to be significant (*p* = 0.031).

**Table 3:** Observed prevalence of FMDV NSP antibodies reported for all the buffalo subspecies tested and filtered by age, year, country and park of collection.

		**No Positive/TOT**	**Observed seroprevalence**	**95% CI**
**Age Group**	Calf (≤6 m)	1/1	100%	-
	Juvenile (>6 m ≤2ys)	3/5	60.0%	22.9% - 88.4%
	Sub-adult (>2ys ≤5ys)	36/51	70.6%	56.9% - 81.4%
	Adult (>5ys)	78/125	62.4%	53.6% - 70.4%
**Year**	1999	30/44	68.2%	53.4% - 80.1%
	2000	29/55	52.7%	39.8% - 65.3%
	2001	13/22	59.1%	38.7% - 76.8%
	2002	49/62	79.0%	67.2% - 87.4%
	2003	6/13	46.1%	23.2% - 70.9%
**Subspecie**	Nile Buffalo	115/168	68.4%	61.1% - 75.0%
	West African Buffalo	12/24	50.0%	31.4% - 68.6%
	African Forest Buffalo	0/4	0%	-
**Country**	Benin	8/18	44.4%	24.5% - 66.3%
	Burkina Faso	4/5	80.0%	36.0% - 98.0%
	Nigeria	0/1	0%	-
	**West Africa**	12/24	50.0%	31.4% - 68.6%
	Central African Republic	52/81	64.2%	53.3% - 73.8%
	Chad	30/53	56.6%	43.3% - 69.1%
	Democratic Republic of Congo	33/34	97.1%	83.8% - 100%
	Gabon	0/4	0%	-
	**Central Africa**	115/172	66.9%	59.5% - 73.5%
**Park**	Pendjari National Park	8/18	44.4%	24.5% - 66.3%
	Pama Reserve	3/3	100%	-
	Arly National Park	1/2	50.0%	9.4% - 90.5%
	Borgu Game Park	0/1	0%	-
	Manovo-Gounda St. Floris National Park	15/19	78.9%	56.1% - 92.0%
	Zemongo Faunal Reserve	6/7	85.7%	46.6% - 99.5%
	Bamingui-Bagoran National Park	3/6	50.0%	18.8% - 81.2%
	Zakouma National Park	22/41	53.7%	38.7% - 67.9%
	Garamba National Park	33/34	97.1%	83.8% - 100%
	Loango National Park	0/4	0%	-

Adjusted-Wald test for age group F_(3, 178)_ = 0.72 (*p* = 0.542).

Adjusted-Wald test for year F_(4, 192)_ = 3.03 (*p* = 0.019).

Adjusted-Wald test for subspecie F_(2, 194)_ = 3.52 (*p* = 0.031).

Adjusted-Wald test for country F_(6, 190)_ = 6.62 (*p* = 0.000).

Adjusted-Wald test for region F_(1, 195)_ = 2.51 (*p* = 0.115).

Adjusted-Wald test for park F_(9, 126)_ = 5.27 (*p* = 0.000).

The age mean of FMD seropositive individuals was estimated to be 9.1 ± 5.2 years with high FMD prevalence values described in those animals aged between 2 and 10 years. However, no effect (*p* = 0.542) of age on the FMD seropositivity levels extracted from each of the categories was observed (Table 3), even though sub-adult and adult categories were those reporting high FMD prevalence and narrow interquartile range, which did not include PI values below the cut-off point. No significant difference between sexes was observed (*p* = 0.23).

Differences in seroprevalence estimates between sampling years were found to be statistically significant (*p* = 0.019). Overall, high level of FMD prevalence was reported in samples collected during 1999 (68.2%) and 2002 (79.0%), although observing a higher seroprevalence for West Africa in samples collected in 2000 (80.0%) as opposed to the seroprevalence (83.9%) reported for Central Africa in 2002.

No significant difference (*p* = 0.115) resulted for the regional prevalence distribution of FMD. Within each region, high FMD seroprevalence was found in Burkina Faso (80.0%) for West Africa, and in the Democratic Republic of Congo (97.1%) and Central Africa Republic (64.2%) for Central Africa (Table 3).

Considering the area of origin, presence of high levels of antibodies against the NSPs was reported in those buffalo populations resident in the Garamba National Park (97.1%) of the Democratic Republic of Congo, the Zemongo Reserve (85.7%) and the Manovo-Gounda St. Floris National Park (78.9%) of the Central African Republic (Table 3).

### FMD spatial distribution in buffalo

According to the GLM analysis (Table 4), five main effect variables had statistically detectable association with the FMD positive status to the NS ELISA test observed for buffalo samples. Increased risk in the probability of FMD seropositivity was associated with the longitude (OR = 1.14, *p* = 0.011) of the sample locations, whereas a decrease in risk was reported according to the latitude (OR = 0.79, *p* = 0.000), year (OR = 0.72, *p* = 0.000) and park area (OR = 0.68, *p* = 0.009) variables entered in the model. The kernel smoothed intensity map produced using the predicted FMD prevalence is shown in Figure 5. The areas with high risk of FMD amongst the buffalo populations sampled, as predicted by the model, are located mainly in the bordering areas between south-west Chad and north-west Central African Republic, and in the north-east Democratic Republic of Congo that borders with South Sudan. This spatial range overlaps with the extent of the Aouk and Zakouma National Parks and the Aouk Aoukale Faunal Reserve in Chad, the Manovo-Gounda St. Floris and Bamingui-Bangoran National Parks in the Central African Republic. The Manouvo-Gounda St. Floris and the Zakouma National Parks constitute the same ecological area and, as evidenced by the model prediction and the prevalence reported, it is likely to be regarded as high risk of FMD.

**Table 4:** Generalised linear model (*logit* link) reporting the ORs with corresponding 95% CI for risk factors associated with the FMD seroprevalence reported for all the buffalo subspecies tested.

	**β [95%CI]**	**SE**	**Z**	**p**	**Odds Ratio [95%CI]**
Intercept	4.11 [1.57 – 6.66]	1.23	3.16	0.002	-
Park (Km^2^)	−0.38 [−0.67 – −0.97]	0.15	−2.63	0.009	0.68 [0.51 – 0.91]
Longitude	0.13 [0.03 – 0.23]	0.05	2.53	0.011	1.14 [1.03 – 1.26]
Latitude	−0.23 [−0.37 – −0.10]	0.07	−3.50	0.000	0.79 [0.69 – 0.9]
Year	−0.32 [−0.48 – −0.17]	0.08	−4.10	0.000	0.72 [0.62 – 0.84]
Age	0.01 [0 – 0.01]	0.002	4.31	0.000	1.01 [1 – 1.01]

log-likelihood = −479.58; AIC = 973.17.

**Figure 3:**
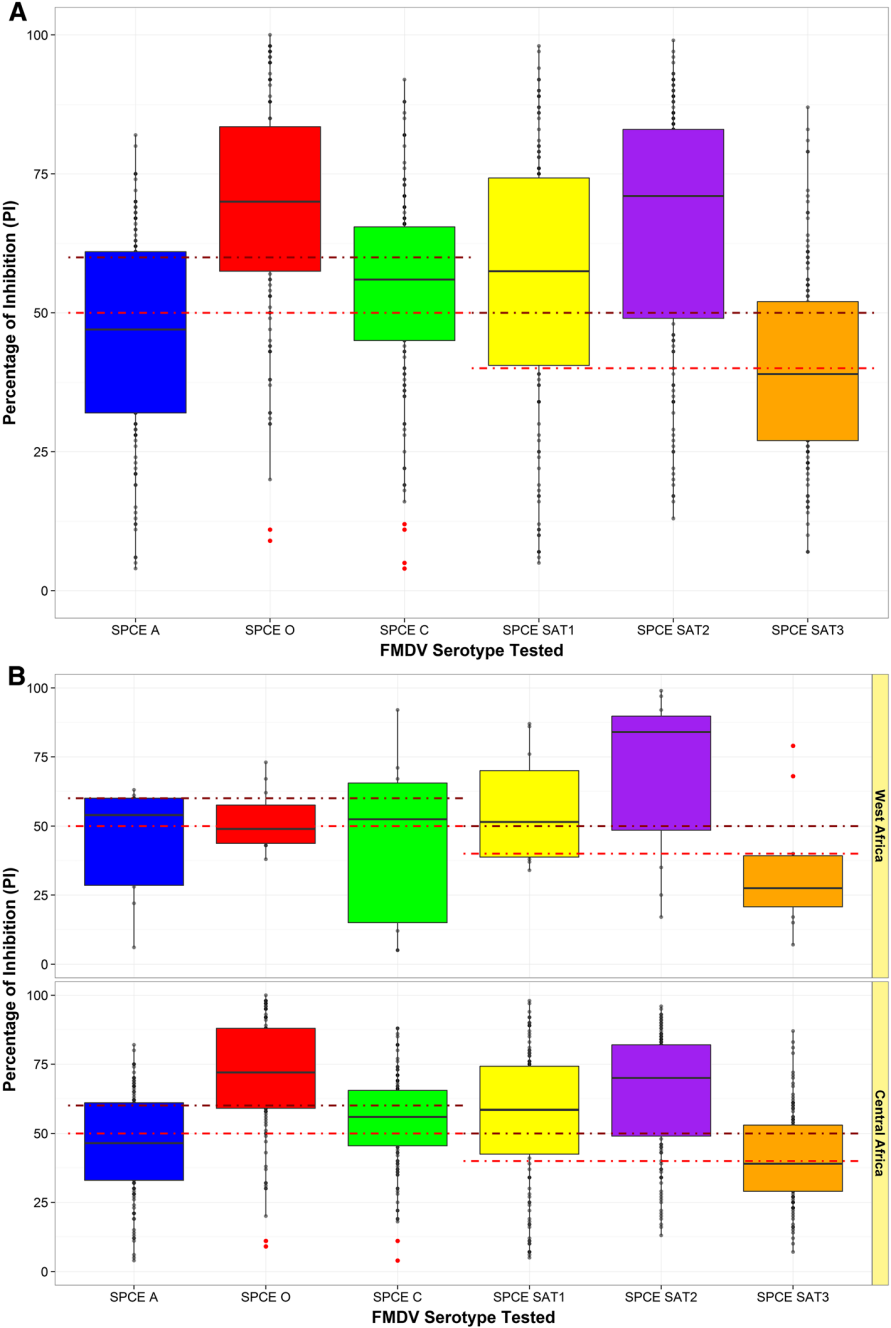
**Box plot of the SPCE percentage of inhibition values for buffalo samples according to each of the FMDV serotypes tested [Overall (A) and regional (B) data].** Cut-off was set at either a PI value of ≥50 or ≥60 for A, O and C serotypes, and at either a PI value of ≥40 or ≥50 for SATs serotypes (red dotted lines) outlier.

### FMDV serotyping profile in buffalo: descriptive and correlation analyses

The overall results from the SPCE analysis of buffalo samples showed presence of antibodies against all the 6 FMDV serotypes tested (Figure 3), with high levels estimated for O (82.3%), SAT 2 (81.9%) and SAT 1 (73.2%) serotypes (Table 5). Increasing the cut-off as described in the methodology section did not largely change the seroprevalence patterns found for O, SAT 1 and SAT 2 FMDV serotypes, differently from the A, C and SAT 3 seropositivities which were reduced to the order of 50%. In addition, 41 out of 102 positive samples (40.2%), 44/104 (42.3%) and 19/93 (20.4%) returned PI values ≥80 for type O, SAT 2 and SAT 1, respectively.

**Figure 4:**
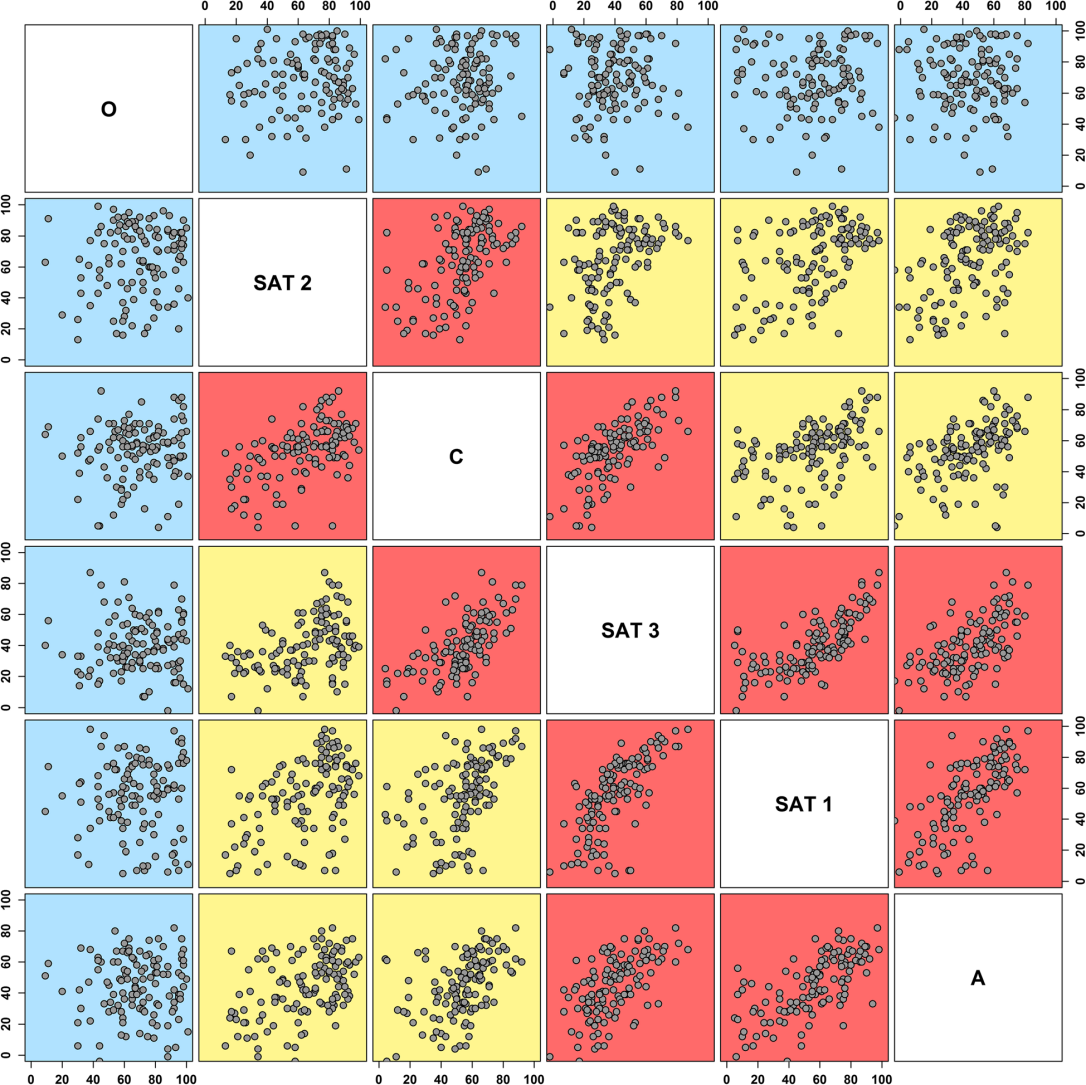
**Scatterplot matrix of the pairwise correlation analysis estimated between PI values obtained from buffalo samples tested for each of the FMDV serotypes by SPCE.** Variables are reordered and coloured according to the returned Pearson correlation values [blue (ρ ≤ 0.3); yellow (0.3 >ρ ≤ 0.5); red (ρ ≥ 0.5)], where higher correlated variables are plot near the diagonal.

**Table 5:** Observed prevalence of serotype-specific FMDV antibodies reported for all the buffalo subspecies tested as overall result and by region of collection.

	**Serotype**	**No Positive/TOT**	**Observed seroprevalence** ^**†**^	**95%CI**
**West Africa**	A	6/12	50.0%	25.4% - 74.6%
	O	6/12	50.0%	25.4% - 74.6%
	C	7/12	58.3%	31.9% - 80.7%
	SAT 1	8/12	66.7%	38.8% - 86.4%
	SAT 2	9/12	75.0%	46.1% - 91.7%
	SAT 3	3/12	25.0%	8.3% - 53.8%
**Central Africa**	A	50/115	43.4%	34.8% - 52.6%
	O	96/112	85.7%	77.9% - 91.1%
	C	77/115	67.0%	57.9% - 74.9%
	SAT 1	85/115	73.9%	65.2% - 81.1%
	SAT 2	95/115	82.6%	74.6% - 88.5%
	SAT 3	56/115	48.7%	39.7% - 57.7%
**TOT**	A	56/127	44.1%	35.8% - 52.8%
	O	102/124	82.3%	74.5% - 88.0%
	C	84/127	66.1%	57.5% - 73.8%
	SAT 1	93/127	73.2%	64.9% - 80.2%
	SAT 2	104/127	81.9%	74.2% - 87.7%
	SAT 3	59/127	46.5%	38.0% - 55.1%

^†^Cut-off values set as ≥50 for A, O and C serotypes, and ≥40 for SATs serotypes.

Interestingly, the pattern of FMD prevalence for each of the 6 serotypes tested was shaped differently according to the region of collection. Although high level of antibody responses against both SAT 1 and SAT 2 FMDV serotypes were recorded for both West and Central Africa, higher prevalence of type O, C and SAT 3 were found in samples collected from Central African countries. Moreover, considering the distribution of PI values returned for each of the 6 serotypes by region (Figure 3), the third quartile (*Q*_3_) of type A, O, SAT 3 and to some extent of C results obtained for the West Africa samples was set below the threshold values and the data distributions of type A and O were largely right-skewed (with most of the data lying below the cut-off points), in contrast with what reported for Central Africa.

Different patterns of reaction to the 6 FMDV serotypes tested were recorded, from sera only positive for a single serotype to multiple reactivities. A number of sera with the highest serotype-specific responses (i.e. highest PI values) were identified for type O (16.7%, 2/12), C (16.7%, 2/12) and SAT 2 (58.3%, 7/12) in samples collected from West Africa, and for type A (2.6%, 3/115), O (47.8%, 55/115), C (5.2%, 6/115), SAT 1 (14.8%, 17/115), SAT 2 (27.0%, 31/115) in those retrieved from Central Africa (Table 6). No sera with the highest serotype-specific response for SAT 3 were reported, even though PI values of up to 79 and 87 were estimated from samples of West and Central Africa, respectively. The potential cross-reaction between pairs of serotypes tested was then assessed computing the pairwise correlation matrix of continuous data (PI values) for all the samples analysed (Figure 4). Statistically significant correlations (*p* = 0.000) with high ρ coefficients were reported for the A–SAT 1 (0.66), A–SAT 3 (0.61), SAT 1–SAT 3 (0.70), C–SAT 3 (0.67) and C–SAT 2 (0.54) pairs. No correlation was found between O and any of the other FMDV serotypes tested (ρ≤ 0.1; *p* > 0.05). The random sample (*n* = 43) extracted from the SPCE positive data was confirmed by the VNT test, which reported positive results at the highest titre of 1:90, 1:178, 1:256, 1:1024 and 1:355 for O, C, SAT 1, SAT 2 and SAT 3 FMDV serotypes, respectively. Inconclusive results were obtained for type A (titre of 1:22).

**Table 6:** Number of buffalo sera with highest serotype-specific FMDV antibodies response [highest PI value] per serotype tested positive on the SPCE by country of collection.

		**A**	**O**	**C**	**SAT 1**	**SAT 2**	**SAT 3**
Benin		0/8 [60]	2/8 [73]	2/8 [92]	0/8 [87]	3/8 [92]	0/8 [79]
Burkina Faso		0/4 [63]	0/4 [67]	0/4 [71]	0/4 [76]	4/4 [99]	0/4 [40]
	**West Africa**	0/12 [63]	2/12 [73]	2/12 [92]	0/12 [87]	7/12 [99]	0/12 [79]
Central African Republic		1/52 [80]	22/52 [95]	4/52 [85]	11/52 [98]	13/52 [93]	0/52 [87]
Chad		2/30 [75]	9/30 [85]	2/30 [74]	2/30 [87]	13/30 [96]	0/30 [81]
Democratic Republic of Congo		0/33 [82]	24/33 [108]	0/33 [88]	4/33 [97]	5/33 [90]	0/33 [83]
	**Central Africa**	3/115 [82]	55/115 [108]	6/115 [88]	17/115 [98]	31/115 [96]	0/115 [87]

**Figure 5:**
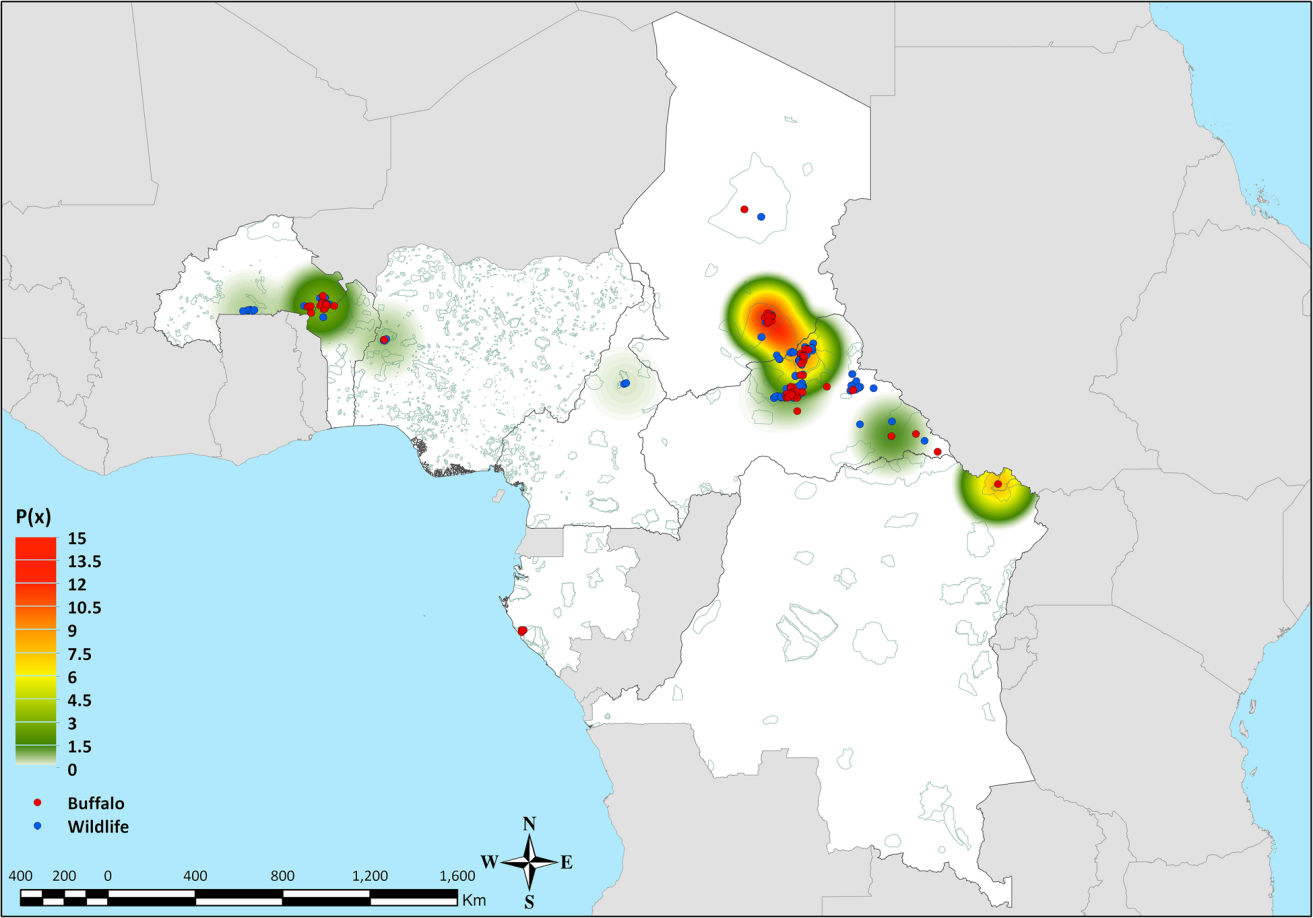
**Kernel density map of the predicted probability μ of FMD seropositivity in buffalo as estimated from the generalised linear model (** ***logit*** **link).** Geographical extent of African wildlife protected areas are sourced and adapted from [69].

## Discussion

This study reports the FMD serological profile extracted from wildlife populations inhabiting the rangelands of West and Central Africa. The results confirm that FMDV circulates within wildlife-livestock ecosystems present in the study regions and in particular in buffalo subspecies, also suggesting that multiple FMDV serotypes may be involved with type O, SAT 2 and SAT 1 being dominant. A different pattern of FMD prevalence for each of the serotypes tested was reported between West and Central Africa, with high levels of serotype-specific antibodies against type O, C and SAT 3 found in buffalo samples sourced from Central Africa. These results would indicate a distinct geographical extent of FMDV serotypes circulating in buffalo populations present in West and Central Africa, which might be associated with transboundary movements of FMDV lineages and, thus, in line with what has been historically described for the FMDV pools 4 and 5 [35,36]. Although historical data of FMDV isolates recovered from buffalo samples within the countries under study at the time of the sampling were generally not available, the here defined FMD distribution in wild ruminants of West and Central Africa might largely contribute in providing a clearer picture of the FMD burden in those largely unstudied regions of sub-Saharan Africa. The reported outbreaks affecting livestock of West Africa since 2000 were caused by FMDV types A, O and SAT 2 circulating in Benin (O and A in 2010), Burkina Faso (O in 2002), Cameroon (O in 2000 and 2005; A and SAT 2 in 2000, 2005 and 2012–13), Mali (O in 2004–05; A in 2004 and 2006), Mauritania (O in 2000–01; A in 2006), Niger (O in 2001 and 2005; SAT 2 in 2007–08 and 2011–12), Senegal (O in 2001 and 2006; SAT 2 in 2009), Togo (O in 2004–05; A in 2005) [37-43]. From 1970, reports of FMD activities in Central Africa were only available for types A and SAT 2 in Chad (1973 and 1972, respectively) and for the Democratic Republic of Congo (O in 2006 and 2010; A in 2011; SAT 2 in 1974, 1979 and 1982) [44]. FMDV type C has never been reported in West and Central Africa and, moreover, up until 2004 this FMDV serotype was only confined to East Africa in Kenya (1957–2004), Ethiopia (1957–1983) and Uganda (1970–71), from when it seems to have been extinct [45]. A very striking results provided from this study is the relatively high proportion (84/127) of serotype C positive sera, although with only 6 producing very high PI values. This may either indicate a potential cross-reactivity with other serotypes (high correlations were found between C and both SAT 2 and SAT 3) or that serotype C may have been circulating without being detected, with the latter less plausible. The SAT 3 serotype has been mainly documented in Southern African countries with occasional isolations in Uganda (1970 and 1997) from samples collected from Cape Buffalo in the southern part of the Queen Elizabeth National Park [46,47]. In 2013, FMDV SAT 3 was isolated from a sub-clinically (or persistently) infected Ankole calf at Nyakatonzi (Kasese District), in close proximity to the northern part of the Queen Elizabeth National Park [48]. These finding may indicate that this serotype is also potentially maintained in buffalo populations present in wildlife ecosystems of Eastern Africa [17].

The SPCE test used in this study for the qualitative and quantitative detection of antibodies against the FMDV serotypes has previously proven to be more robust and specific, and equally sensitive to the Liquid Phase Blocking ELISA (LPBE) [26], but not totally unaffected by serological cross-reactivity between FMDV serotypes, thus representing a valid, easy and fast to process alternative to the VNT. It should be noted that the SPCE has not been validated for testing wildlife sera, but only for cattle, pigs and sheep [26,27], and this might have an impact on the results here generated. However, the selection of higher cut-off values for the SPCE positivity threshold, aiming at reducing a potential false positive response, did not change the seroprevalence figures here reported and, furthermore, several buffalo samples returned PI values of ≥80 thus indicating presence of high antibody levels. In addition, a random sample of the positive sera generated from the SPCE has been confirmed by VNT testing. The mismatch between the strains used for the VNT testing and antibodies present in the sera might have an impact on the results obtained. However, the precise selection of an appropriate test antigen would not be possible when testing sera of unknown status and, therefore, it has been assumed that antigenic differences were limited, with positive samples still producing high titres to the specific strains than to the other serotypes. Although correlations between serotype-specific responses have been evaluated, the extent to which findings of individual sera reactive to multiple serotypes is due to serological cross-reactivity or multiple infections has not been entirely ruled out. However, subsequent multiple serotypes infections do occur and even simultaneous multiple infection cannot be excluded. In a previous study it has been demonstrated that buffalo carriers are refractory to reinfection with the same strain of virus [49,50] thus supporting the hypothesis of potential co-infections and/or subsequent infections with more than one serotypes. Although the results here reported suggest infections of buffalo subspecies with different FMDV serotypes, there is little published on non-SAT serotypes in buffalo population [19-23], with no evidences supporting the hypothesis that domestic types of FMDV has come from buffalo as carrier. In a recent study, the phylogenetic descent of the SAT 2 serotype across the entire African continent was regarded to have originated from a FMDV ancestor formerly infecting Cape buffalo, also evaluating that interspecies virus transitions might occur between Cape buffalo and cattle, and vice versa [51]. Although this would support the hypothesis of FMDV shifting between wildlife and livestock, the incomplete and biased nature of the analysed data could potentially derive confounding results. Nevertheless, genetic and epidemiological analyses have clearly shown on specific occasions the close relationship between SATs viruses infecting buffalo and other wild species (e.g. Impala) to those causing outbreaks in cattle [16,52,53]. In addition, a field study conducted in Ethiopia found significant association between cattle exposed to FMDV and their contact history with wildlife [54], whilst a recent study conducted at the periphery of protected areas in Zimbabwe indicates that interactions between livestock and buffalo populations can account for FMD primary outbreaks [55]. Besides studies based on serological investigations, to what extent types of FMDV prevalent in domestic ruminants infect wildlife is unknown; hence, until this issue is investigated thoroughly it will constitute a major deficiency in understanding the epidemiology of FMD in large parts of sub-Saharan Africa. Therefore, further field studies are warranted to collect clinical samples, in order to enable the genome characterisation of FMDV lineages circulating within wild animal species of West and Central Africa and to confirm if FMDV serotypes normally present in domestic livestock are really mixing with buffalo and, thus, eventually become established in those populations. However, it should be noticed that two significant buffalo populations at present exist in West and Central Africa, which are susceptible to share cattle grounds (Figures 1 and 5): the W-Arly-Pendjari (WAP) Parks Complex (tranfrontier area shared between Benin, Burkina Faso and Niger, which hosts around 10000+ buffalo) and the Zakouma National Park (which hosts around 7000+ buffalo). This geographic distribution of buffalo would therefore likely contribute to reduce the mixing between buffalo and livestock thus decreasing the risk of FMD transmission.

Wild animal species already reported to be susceptible to the FMDV [7] were confirmed in this study. In addition, presence of antibodies against the FMDV has not been previously described in Buffon’s Kob (*Kobus kob*) and Oribi (*Ourebia ourebi*), therefore increasing the number of wild ruminant species reported to be susceptible to FMD infection in sub-Saharan Africa. In addition, the FMD prevalence found in non-buffalo species of West and Central Africa is higher than what has previously been reported in other studies targeting wildlife ecosystems of Eastern Africa and Zimbabwe [17,18,56]. The high FMD prevalence found in Bohor Reedbuck and Waterbuck might reflect their ecology and living ecosystem: in fact they are dependent on water with living habitats close to water sources, which might indicate a link between FMD transmission within and between wildlife species (and/or between domestic and wild animals) when congregating at watering points. This study provides the first evidence of FMDV exposure in subspecies of the *Syncerus* genus other than the Cape buffalo [7], thus accounting for their potential role in the epidemiology of FMD outside the living habitat of Cape buffalo in Eastern and Southern African regions. Although the predicted spatial distribution of FMD in buffalo might reflect the epidemiological status at the time of the sampling, a generalisation to the current situation might be expected since no effective control measures have been implemented in recent years either in West or Central African regions, and up-to-date data have not been published. In addition, although accounting for the extent of the protected areas present in West and Central Africa, the predicted FMD spatial distribution was not intended to resolve the landscape structure of the study regions, and so this might cover areas where buffalo ecological niches might be absent. Therefore, a more extensive sampling frame would be required to provide a more exhaustive indication of the spatial burden of FMD in wildlife ecosystems of West and Central Africa. This initial attempt would, nevertheless, provide useful information to be linked for a broad scale strategic FMD monitoring planning.

According to the buffalo subspecies tested, the FMD prevalence found might reflect the different social organization and population biology of each of the subspecies considered. For example, the African forest buffalo herds are quite isolated, have a small animal density (herd size of about 3–25 individuals) and move in a limited home-range (~2.3–8 km^2^) compared to the Cape buffalo. In addition, they have a very secretive behaviour and a limited distribution, mainly found in rainforest ecosystem [57]. Savannah buffalo (West African and Nile buffalo) more frequently split into smaller herds, herd-switching is more common and they have a large home range. This is opposed to Cape buffalo herds that are more densely populated (e.g. mass herds can vary from few hundreds up to several thousand individuals) and engage in long-distance dispersal [58]. In a previous study conducted in Cape buffalo population of East Africa, a 67.7% of the total samples tested was reported as FMD positive [17]. Therefore, this might indicate different patterns of FMD susceptibility across the different buffalo subspecies that might be linked to the species ecology, even though only four African forest buffalo samples were available for testing. In fact, susceptibilities to the FMDV of buffalo living in savannah habitat are consistent across sub-species but African forest buffalo might show a different epidemiology. On the other hand, this could be a concurrent result of the host living habitat, the social behaviour of each of the buffalo subspecies, and the extent of the FMD geographical distribution (i.e. the African forest buffalo samples were collected from Gabon, in which FMD has never been reported in the periods between 1996 and 2003 [59], and between 2006 and 2012 [60]). In addition, it should be noted that the African forest buffalo were darted in the Loango National Park where the livestock presence is very low and especially absent from the darting area, that means lack of contacts between buffalo and cattle besides the very low size of herds (<8 heads). From the model estimates, the odds of a buffalo resulting FMD positive by NS ELISA is reduced by 0.98 for every 10 km^2^ increase in the park area. This would be directly correlated with the density of buffalo population, which might indicate that in large ecosystems herds tend to be sparser, not overlapping their home ranges, and thus diminishing the risk of transmitting and maintaining the disease. In addition, the rainforest area of western and central Africa represents a limiting factor for the spatial and demographic expansion of buffalo herds (and livestock), as would be the case for African forest buffalo that mainly inhabits forest clearings [61]. Figures indicate that 89.3% of rainforests is present in Central Africa, where the Democratic Republic of Congo accounts for the largest African lowland rainforest area (53.6%) [62]. However, the FMD prevalence (97.1%) obtained from the Democratic Republic of Congo was resulting from Nile buffalo samples collected in the Garamba National Park, which mainly covers vast grass savannahs and woodlands. Furthermore, density of buffalo population fluctuates according to seasons, which tend to be reduced during the dry season as at this time of the year the size of the area for grazing and watering is really reduced compared to the rainy season.

Direct contacts between buffalo and livestock seem to be rare, with degrees of variability determined by ecosystem structure and climatic cycles. It seems to be more common in open habitats and with plains species (e.g. during mass migration of wildebeest, topi, zebra and gazelles as in the Mara-Serengeti ecosystem in East Africa) and during the dry seasons, when water availability constrains animals to closely congregate thus increasing the transmission likelihood of water-related infections [63]; in fact, these are also the times of the highest level of residency and density within protected areas of both livestock and buffalo. In addition, it should be noted that wildlife usually avoids livestock and human contacts (i.e. at watering points or locations with key forage resources) in a space-time fashion unless habituated. As FMD requires a relatively close contact setting for interspecies transmission, the FMD interface between wildlife and livestock should, therefore, not be seen as a direct physical interaction but as an indirect contact (i.e. through soil, forage and water contaminated by bodily discharge of infected animals), which might be regarded as the most likely factor to be associated with the risk of FMDV transfer from domestic to wildlife species. Eventually, the spread of FMD within the wildlife-domestic interface might be driven by a complex interplay of risk factors, including biophysical and climatic features, ecological traits and human practices.

The control of the wildlife-livestock interface in the transmission of FMD has been only successfully applied in South Africa at a considerable ecological and economic cost [64,65] by the means of herds separation (e.g. strict land-use policies, animal movement controls and fencing) and buffer vaccination of livestock population around the source of virus [66]. However, in countries where wildlife populations are integrated with extensive nomadic and semi-nomadic pastoralism, as would be the case for West and Central Africa, the risk of FMD spread increases not only for the difficulty in applying effective control measures (e.g. pastoralists usually rely on ethnoveterinary practices [67] and move within and between countries in an uncontrolled fashion), but also for the increase in the land-use pressure and conflict between pastoralists and wildlife competing for grazing and water resources. As example, the St. Floris National Park, Central African Republic, is boasting a large population of buffalo and is a grazing and transhumance crossing land for the Fulani pastoralist tribe. A 78.9% of FMD prevalence in Nile buffalo has been reported in this ecosystem, with high serotype-positive responses against type O, SAT 1 and SAT 2. The demographic growth, the expansion of cultivation (e.g. agro-pastoral systems), the development of local governance on natural resources [65] and the reduction of rangeland resources in Africa have indeed led to increased sharing of resources between domestic and wildlife species and hence the risk of diseases transmission. However, the ecological processes driving the FMDV evolution and transmission in the sub-Saharan African ecosystems still remain poorly documented. In this context the nature of the wildlife-livestock interface in pastoral landscapes and its complex socio-ecological-economic interactions have not been entirely studied and need to be thoroughly investigated. The importance and, at the same time, the difficulties in controlling the FMD in the sub-Saharan Africa relies on the unique diversity and numbers of the wild species present and the explosive growth of the human population, which consequently needs to create solutions that would work for improving the agriculture standards, the sanitary safety of livestock trade, the sustainable land use and the biodiversity conservation of wildlife ecosystems.

## References

[1] Alexandersen S, Zhang Z, Donaldson AI, Garland AJ (2003). The pathogenesis and diagnosis of foot-and-mouth disease. J Comp Pathol.

[2] Thomson GR, Vosloo W, Bastos AD (2003). Foot and mouth disease in wildlife. Virus Res.

[3] Arzt J, Baxt B, Grubman MJ, Jackson T, Juleff N, Rhyan J, Rieder E, Waters R, Rodriguez LL (2011). The pathogenesis of foot-and-mouth disease II: viral pathways in swine, small ruminants, and wildlife; myotropism, chronic syndromes, and molecular virus-host interactions. Transbound Emerg Dis.

[4] Dawe PS, Flanagan FO, Madekurozwa RL, Sorensen KJ, Anderson EC, Foggin CM, Ferris NP, Knowles NJ (1994). Natural transmission of foot-and-mouth disease virus from African buffalo (Syncerus caffer) to cattle in a wildlife area of Zimbabwe. Vet Rec.

[5] Bastos A, Boshoff CI, Keet DF, Bangis RG, Thomson GR (2000). Natural transmission of foot-and-mouth disease virus between African buffalo (Syncerus caffer) and impala (Aepyceros melampus) in the Kruger National Park, South Africa. Epidemiol Infect.

[6] Sutmoller P, Thomson GR, Hargreaves SK, Foggin CM, Anderson EC (2000). The foot and mouth disease risk posed by African buffalo within wildlife conservancies to the cattle industry of Zimbabwe. Prev Vet Med.

[7] Weaver GV, Domenech J, Thierman AR, Karesh WB (2013). Foot and mouth disease: a look from the wild side. J Wild Dis.

[8] Vosloo W, Bastos AD, Kirkbride E, Esterhuysen JJ, van Rensburg DJ, Bengis RG, Keet DW, Thomson GR (1996). Persistent infection of African buffalo (Syncerus caffer) with SAT-type foot-and-mouth disease viruses: rate of fixation of mutations, antigenic change and interspecies transmission. J Gen Virol.

[9] Thomson GR, Vosloo W, Esterhuysen JJ, Bengis RG (1992). Maintenance of foot and mouth disease viruses in buffalo (Syncerus caffer Sparrman, 1779) in southern Africa. Rev Sci Tech.

[10] Hedger RS (1972). Foot-and-mouth disease and the African buffalo (Syncerus caffer). J Comp Pathol.

[11] Condy JB, Hedger RS, Hamblin C, Barnett IT (1985). The duration of the foot-and-mouth disease virus carrier state in African buffalo (i) in the individual animal and (ii) in a free-living herd. Comp Immunol Microbiol Infect Dis.

[12] Dawe PS, Sorensen K, Ferris NP, Barnett IT, Armstrong RM, Knowles NJ (1994). Experimental transmission of foot-and-mouth disease virus from carrier African buffalo (Syncerus caffer) to cattle in Zimbabwe. Vet Rec.

[13] Bruckner GK, Vosloo W, Du Plessis BJ, Kloeck PE, Connoway L, Ekron MD, Weaver DB, Dickason CJ, Schreuder FJ, Marais T, Mogajane ME (2002). Foot and mouth disease: the experience of South Africa. Rev Sci Tech.

[14] Vosloo W, Boshoff K, Dwarka R, Bastos A (2002). The possible role that buffalo played in the recent outbreaks of foot-and-mouth disease in South Africa. Ann NY Acad Sci.

[15] Hargreaves SK, Foggin CM, Anderson EC, Bastos AD, Thomson GR, Ferris NP, Knowles NJ (2004). An investigation into the source and spread of foot and mouth disease virus from a wildlife conservancy in Zimbabwe. Rev Sci Tech.

[16] Vosloo W, Thompson PN, Botha B, Bengis RG, Thomson GR (2009). Longitudinal study to investigate the role of impala (Aepyceros melampus) in foot-and-mouth disease maintenance in the Kruger National Park, South Africa. Transbound Emerg Dis.

[17] Bronsvoort BM, Parida S, Handel I, McFarland S, Fleming L, Hamblin P, Kock R (2008). Serological survey for foot-and-mouth disease virus in wildlife in eastern Africa and estimation of test parameters of a nonstructural protein enzyme-linked immunosorbent assay for buffalo. Clin Vaccine Immunol.

[18] Condy JB, Herniman KA, Hedger RS (1969). Foot-and-mouth disease in wildlife in Rhodesia and other African territories. A serological survey. J Comp Pathol.

[19] Ayebazibwe C, Mwiine FN, Balinda SN, Tjornehoj K, Masembe C, Muwanika VB, Okurut AR, Siegismund HR, Alexandersen S (2010). Antibodies against foot-and-mouth disease (FMD) virus in African buffalos (Syncerus caffer) in selected National Parks in Uganda (2001–2003). Transbound Emerg Dis.

[20] Ayebazibwe C, Mwiine FN, Tjornehoj K, Balinda SN, Muwanika VB, Ademun Okurut AR, Belsham GJ, Normann P, Siegismund HR, Alexandersen S (2010). The role of African buffalos (Syncerus caffer) in the maintenance of foot-and-mouth disease in Uganda. BMC Vet Res.

[21] Kalema-Zikusoka G, Bengis R, Michel A, Woodford M (2005). A preliminary investigation of tuberculosis and other diseases in African buffalo (Syncerus caffer) in Queen Elizabeth National Park, Uganda. Onderstepoort J Vet Res.

[22] Hamblin C, Anderson EC, Jago M, Mlengeya T, Hipji K (1990). Antibodies to some pathogenic agents in free-living wild species in Tanzania. Epidemiol Infect.

[23] Anderson EC, Doughty WJ, Anderson J, Paling R (1979). The pathogenesis of foot-and-mouth disease in the African buffalo (Syncerus caffer) and the role of this species in the epidemiology of the disease in Kenya. J Comp Pathol.

[24] Chardonnet P, Kock R (2001). Final report of the African Wildlife Veterinary Project (November 1998 - June 2000).

[25] Sorensen KJ, de Stricker K, Dyrting KC, Grazioli S, Haas B (2005). Differentiation of foot-and-mouth disease virus infected animals from vaccinated animals using a blocking ELISA based on baculovirus expressed FMDV 3ABC antigen and a 3ABC monoclonal antibody. Arch Virol.

[26] Li Y, Swabey KG, Gibson D, Keel PJ, Hamblin P, Wilsden G, Corteyn M, Ferris NP (2012). Evaluation of the solid phase competition ELISA for detecting antibodies against the six foot-and-mouth disease virus non-O serotypes. J Virol Methods.

[27] Paiba GA, Anderson J, Paton DJ, Soldan AW, Alexandersen S, Corteyn M, Wilsden G, Hamblin P, MacKay DK, Donaldson AI (2004). Validation of a foot-and-mouth disease antibody screening solid-phase competition ELISA (SPCE). J Virol Methods.

[28] Golding SM, Hedger RS, Talbot P (1976). Radial immuno-diffusion and serum-neutralisation techniques for the assay of antibodies to swine vesicular disease. Res Vet Sci.

[29] Core Team R (2015). R, A language and environment for statistical computing.

[30] Brown L, Cai T, DasGupta A (2001). Interval estimation for a binomial proportion. Stat Sci.

[31] Koch G, Freeman GDH, Freeman JL (1975). Strategies in the multivariate analysis of data from complex surveys. Int Stat Rev.

[32] Hosmer D, Lemeshow S (2000). Applied logistic regression.

[33] Silverman B (1986). Density estimation for statistics and data analysis.

[34] Kirkwood B, Sterne J (2003). Essential medical statistics.

[35] Di Nardo A, Knowles NJ, Paton DJ (2011). Combining livestock trade patterns with phylogenetics to help understand the spread of foot and mouth disease in sub-Saharan Africa, the Middle East and Southeast Asia. Rev Sci Tech.

[36] Rweyemamu M, Roeder P, Mackay D, Sumption K, Brownlie J, Leforban Y, Valarcher JF, Knowles NJ, Saraiva V (2008). Epidemiological patterns of foot-and-mouth disease worldwide. Transbound Emerg Dis.

[37] Sangare O, Bastos AD, Venter EH, Vosloo W (2003). Retrospective genetic analysis of SAT-1 type foot-and-mouth disease outbreaks in West Africa (1975–1981). Vet Microbiol.

[38] Sangare O, Bastos AD, Venter EH, Vosloo W (2004). A first molecular epidemiological study of SAT-2 type foot-and-mouth disease viruses in West Africa. Epidemiol Infect.

[39] Bronsvoort BM, Radford AD, Tanya VN, Nfon C, Kitching RP, Morgan KL (2004). Molecular epidemiology of foot-and-mouth disease viruses in the Adamawa province of Cameroon. J Clin Microbiol.

[40] Ehizibolo DO, Perez AM, Carrillo C, Pauszek S, AlKhamis M, Ajogi I, Umoh JU, Kazeem HM, Ehizibolo PO, Fabian A, Berninger M, Moran K, Rodriguez LL, Metwally SA (2014). Epidemiological analysis, serological prevalence and genotypic analysis of foot-and-mouth disease in Nigeria 2008–2009. Transbound Emerg Dis.

[41] Fasina FO, Connell DR, Talabi OA, Lazarus DD, Adeleke GA, Olusanya TP, Hernandez JA (2013). Foot-and-mouth disease virus strains and examination of exposure factors associated with seropositivity of cattle herds in Nigeria during 2007–2009. Prev Vet Med.

[42] Gorna K, Houndje E, Romey A, Relmy A, Blaise-Boisseau S, Kpodekon M, Saegerman C, Moutou F, Zientara S, Bakkali Kassimi L (2014). First isolation and molecular characterization of foot-and-mouth disease virus in Benin. Vet Microbiol.

[43] Ludi A, Ahmed Z, Pomeroy LW, Pauszek SJ, Smoliga GR, Moritz M, Dickmu S, Abdoulkadiri S, Arzt J, Garabed R, Rodriguez LL: Serotype diversity of Foot-and-Mouth-Disease virus in livestock without history of vaccination in the far North Region of Cameroon. Transbound Emerg Dis, (in press) 10.1111/tbed.12227 PMC4499489 24735162

[44] Ferris NP, Donaldson AI (1992). The World Reference Laboratory for Foot and Mouth Disease: a review of thirty-three years of activity (1958–1991). Rev Sci Tech.

[45] Roeder PL, Knowles NJ (2008) Foot-and-mouth disease virus type C situation: the first target for eradication? In: FAO (eds) Open Session of the European Commission for the Control of Foot-and-Mouth Disease Standing Technical Committee: 14–17 October 2008; Erice, Italy

[46] Hedger RS, Forman AJ, Woodford MH (1973). Foot-and-mouth disease in East African buffalo. Bull Epizoot Dis Afr.

[47] Bastos AD, Anderson EC, Bengis RG, Keet DF, Winterbach HK, Thomson GR (2003). Molecular epidemiology of SAT3-type foot-and-mouth disease. Virus Genes.

[48] Dhikusooka MT, Tjornehoj K, Ayebazibwe C, Namatovu A, Ruhweza S, Siegismund HR, Wekesa SN, Normann P, Belsham GJ (2015). Foot-and-mouth disease virus serotype SAT 3 in long-horned Ankole calf, Uganda. Emerg Infect Dis.

[49] Hedger RS, Condy JB, Golding SM (1972). Infection of some species of African wildlife with foot-and-mouth disease virus. J Comp Pathol.

[50] Woodbury EL (1995). A review of the possible mechanisms for the persistence of foot-and-mouth disease virus. Epidemiol Infect.

[51] Hall MD, Knowles NJ, Wadsworth J, Rambaut A, Woolhouse ME (2013). Reconstructing geographical movements and host species transitions of foot-and-mouth disease virus serotype SAT 2. MBio.

[52] Vosloo W, Bastos AD, Sangare O, Hargreaves SK, Thomson GR (2002). Review of the status and control of foot and mouth disease in sub-Saharan Africa. Rev Sci Tech.

[53] Vosloo W, Bastos ADS, Sahle M, Sangare O, Dwarka RM, Osofsky SA, Cleaveland S, Karesh WB, Kock MD, Nyhus PJ, Starr L, Yang A (2005). Virus topotype and the role of wildlife in foot and mouth disease in Africa. Conservation and development interventions at the wildlife/livestock interface: implications for wildlife, livestock and human health.

[54] Molla B, Ayelet G, Asfaw Y, Jibril Y, Ganga G, Gelaye E (2010). Epidemiological study on foot-and-mouth disease in cattle: seroprevalence and risk factor assessment in South Omo zone, south-western Ethiopia. Transbound Emerg Dis.

[55] Miguel E, Grosbois V, Caron A, Boulinier T, Fritz H, Cornelis D, Foggin C, Makaya PV, Tshabalala PT, De Garine-Wichatitsky M (2013). Contacts and foot and mouth disease transmission from wild to domestic bovines in Africa. Ecosphere.

[56] Anderson EC, Foggin CM, Atkinson M, Sorensen KJ, Madekurozwa RL, Nqindi J (1993). The role of wild animals, other than buffalo, in the current epidemiology of foot-and-mouth disease in Zimbabwe. Epidemiol Infect.

[57] Melletti M, Penteriani V, Mirabile M, Boitani L (2007). Some behavioral aspects of forest buffalo (syncerus caffer nanus): from herd to individual. J Mammal.

[58] Prins HHT (1996). Ecology and behaviour of the African buffalo.

[59] World Organisation for Animal Health: Handistatus II - OIE Animal Health Information Database. http://web.oie.int/hs2/report.asp?lang=en. Accessed 8 June 2015.

[60] World Organisation for Animal Health: WAHID Interface - OIE World Animal Health Information Database. http://www.oie.int/wahis_2/public/wahid.php/Wahidhome/Home. Accessed 8 June 2015.

[61] Melletti M, Penteriani V, Boitani L (2007). Habitat preferences of the secretive forest buffalo (Syncerus caffer nanus) in Central Africa. J Zool.

[62] Malhi Y, Adu-Bredu S, Asare RA, Lewis SL, Mayaux P (2013). African rainforests: past, present and future. Philos Trans R Soc Lond B Biol Sci.

[63] Bengis RG, Kock RA, Fischer J (2002). Infectious animal diseases: the wildlife/livestock interface. Rev Sci Tech.

[64] Kock R, Kock M, de Garine-Wichatitsky M, Chardonnet P, Caron A, Melletti M, Burton J (2014). Livestock and buffalo (Syncerus caffer) interfaces in Africa: ecology of disease transmission and implications for conservation and development. Ecology, Evolution and Behaviour of Wild Cattle Implication for Conservation.

[65] Ferguson KJ, Cleaveland S, Haydon DT, Caron A, Kock RA, Lembo T, Hopcraft JG, Chardonnet B, Nyariki T, Keyyu J, Paton DJ, Kivaria FM (2013). Evaluating the potential for the environmentally sustainable control of foot and mouth disease in Sub-Saharan Africa. Ecohealth.

[66] Thomson GR (1995). Overview of foot and mouth disease in southern Africa. Rev Sci Tech.

[67] Ole-Mairon JO (2003). The Maasai ethnodiagnostic skill of livestock diseases: a lead to traditional bioprospecting. J Ethnopharmacol.

[68] IUCN: Syncerus caffer. In: IUCN Red List of Threatened Species Version 2012.1. 2012. http://iucnredlist.org Accessed 25 February 2014.

[69] IUCN UNEP-WCMC, IUCN, UNEP-WCMC (2014). The World Database on Protected Areas (WDPA) [On-line].

